# Supplementary material for: Association of Root Hair Length and Density with Yield-Related Traits and Expression Patterns of TaRSL4 Underpinning Root Hair Length in Spring Wheat
Source: Plants (Basel). 2022 Aug 29;11(17):2235. doi: 10.3390/plants11172235 (PMC9460385; doi:10.3390/plants11172235)
Supplement: Supplementary file 1 [file plants-11-02235-s001.zip › Supplementary Figures .pdf]

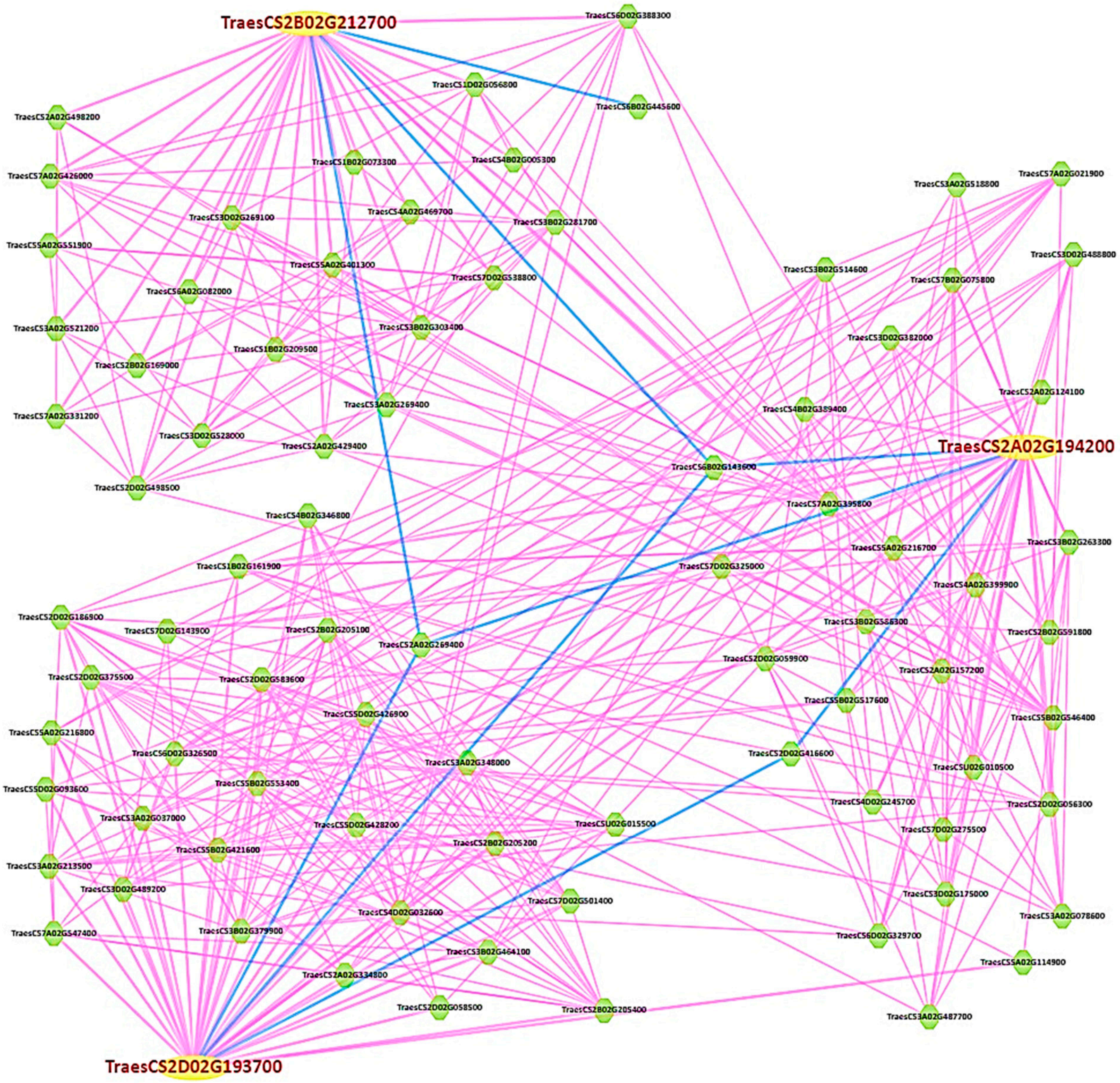

Figure S1. Global RNA-seq network of *TaRSL4* and its co-expressed genes. Interaction lines in pink indicate positive co-expression relationship with target protein whereas blue lines indicate negative co-expression relationship with target protein.

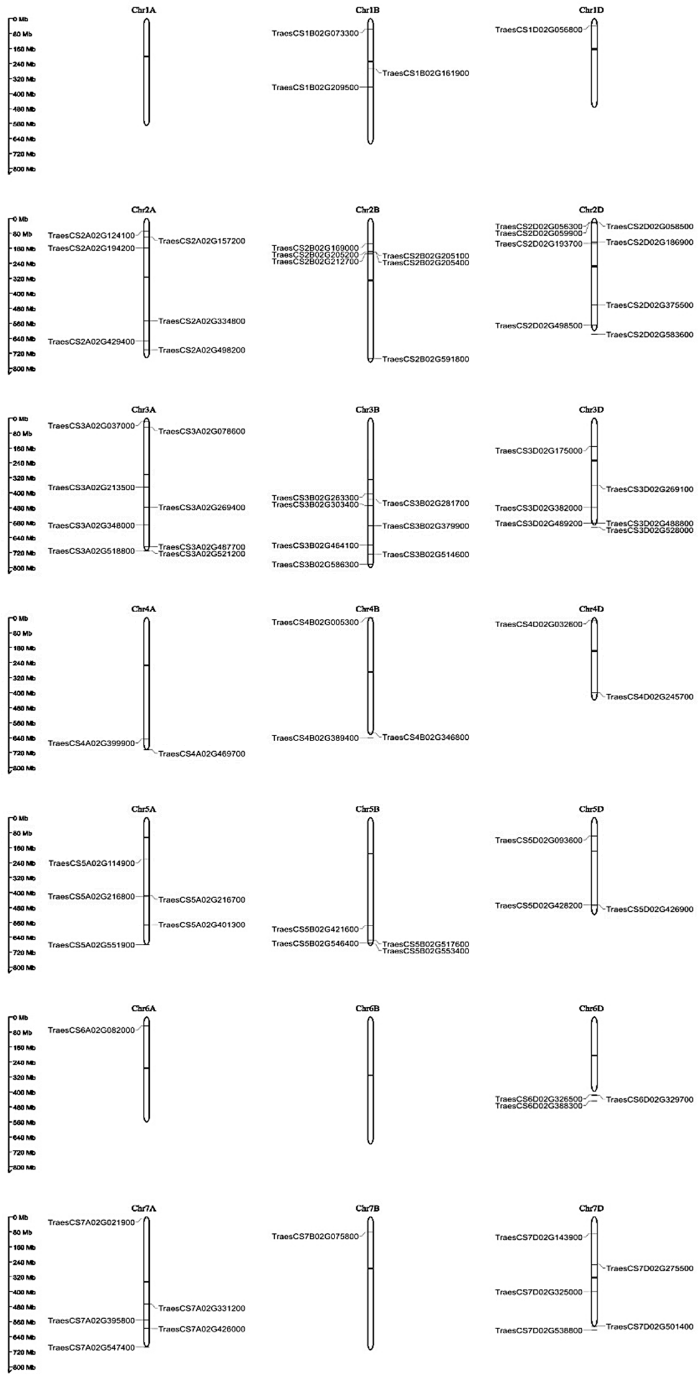

Figure S2. Chromosome location of genes co-expressed with *TaRSL4* under Global RNA-seq network.

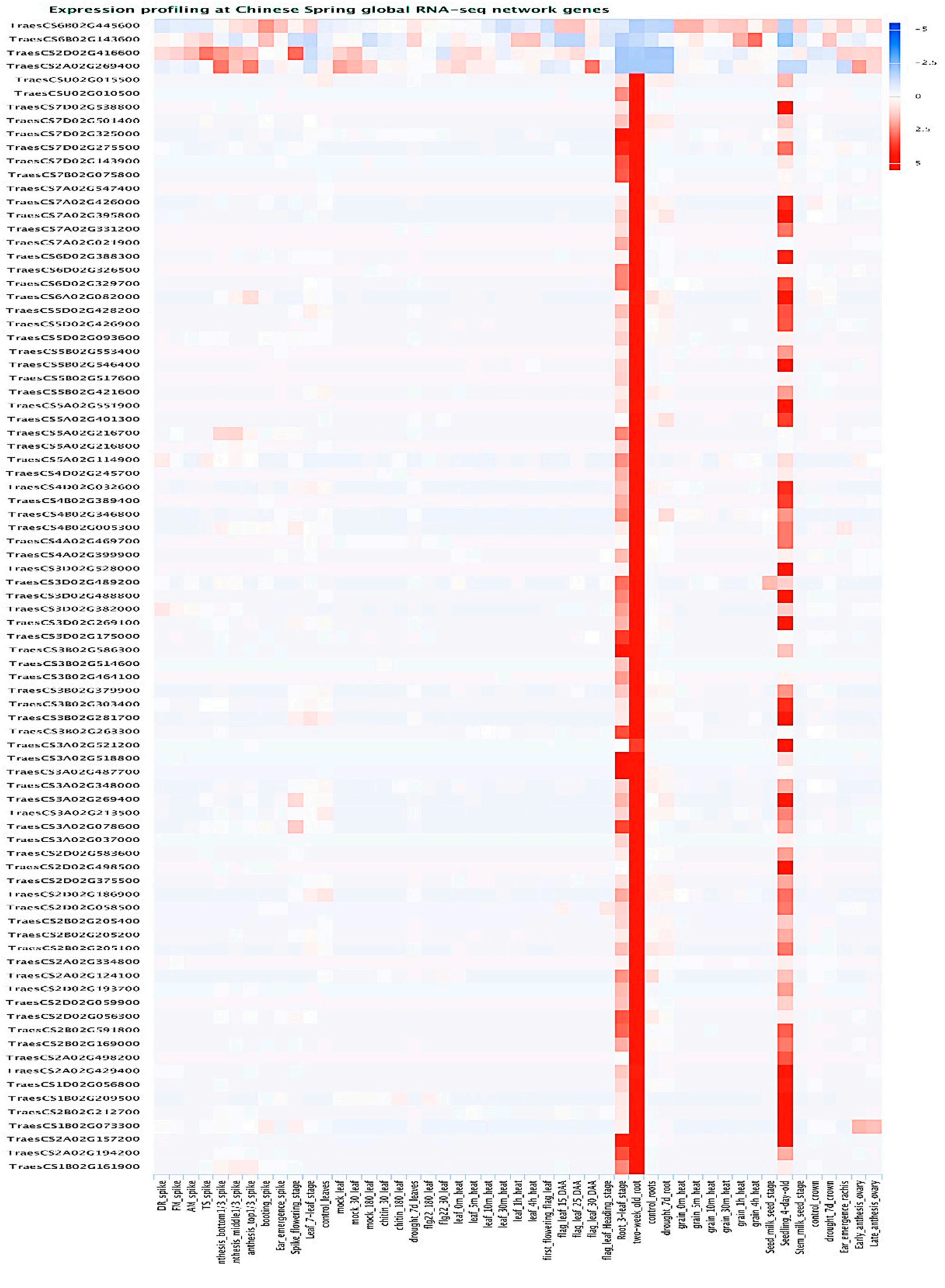

Figure S3. Expression profiling of co-expressed genes of global RNAseq network of Chinese Spring at various growth stages.

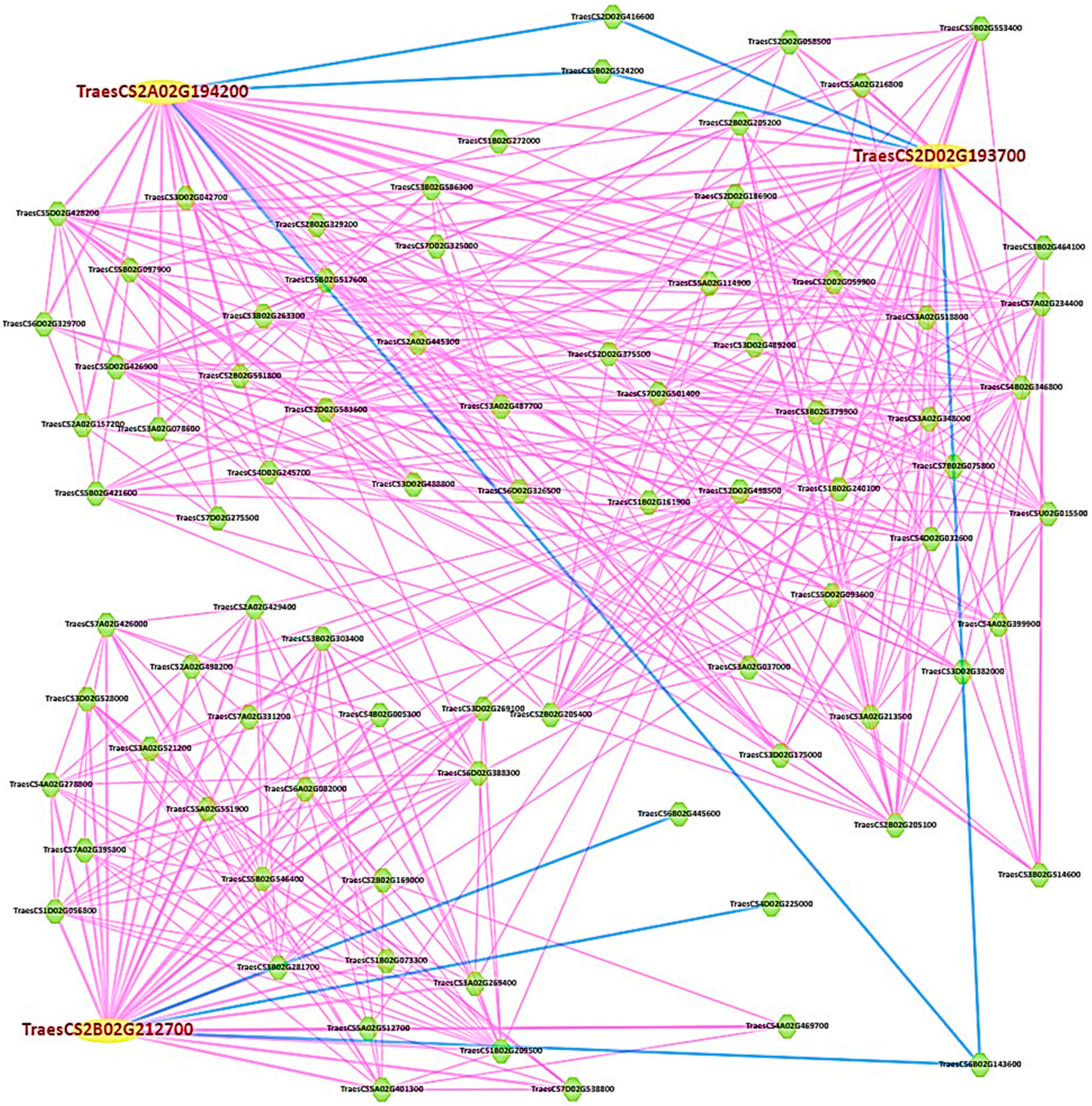

Figure S4. Tissue-specific RNA-seq network of *TaRSL4* and its co-expressed genes. Interaction lines in pink indicate positive co-expression relationship with target protein whereas blue lines indicate negative co-expression relationship with target protein.

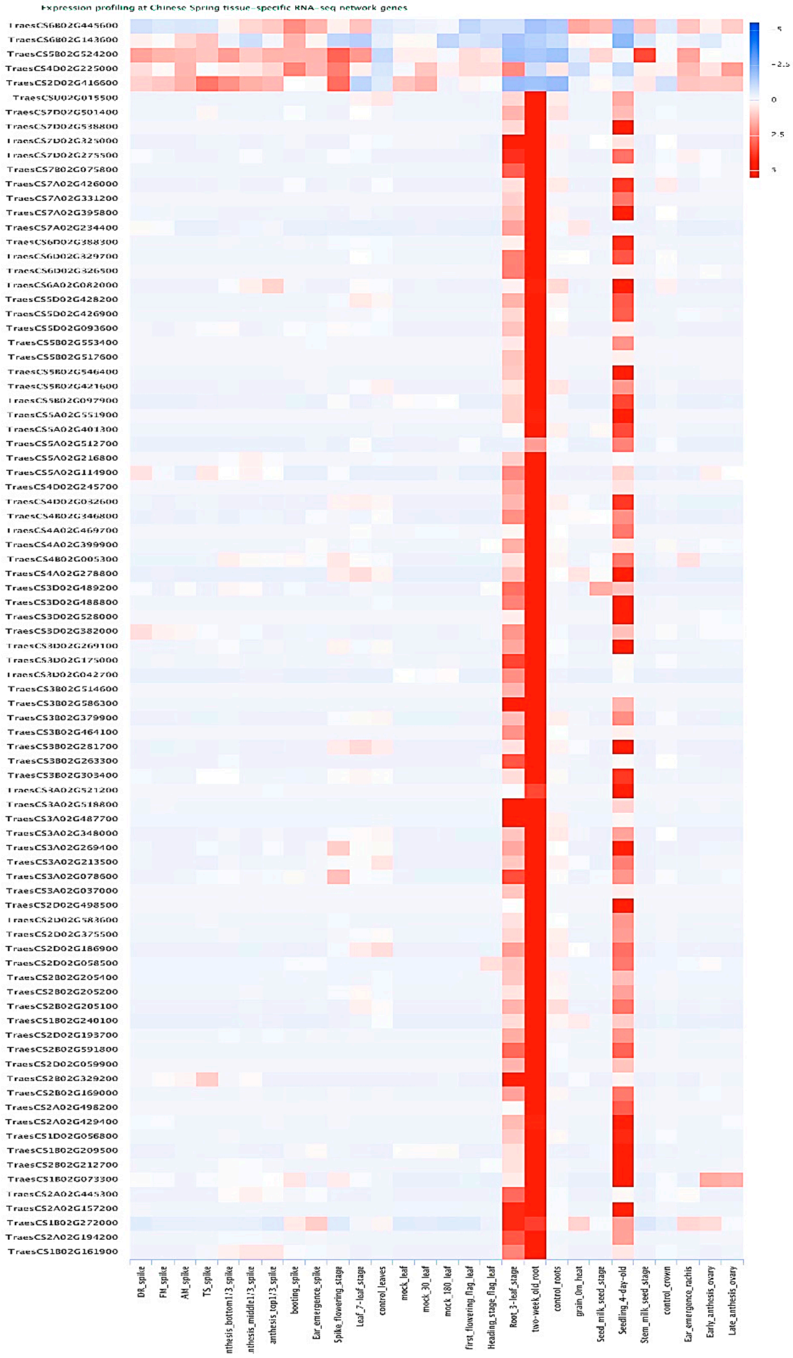

Figure S5. Expression profile of tissue-specific RNA-seq network of *TaRSL4* and its co-expressed genes in Chinese Spring at various growth stages.

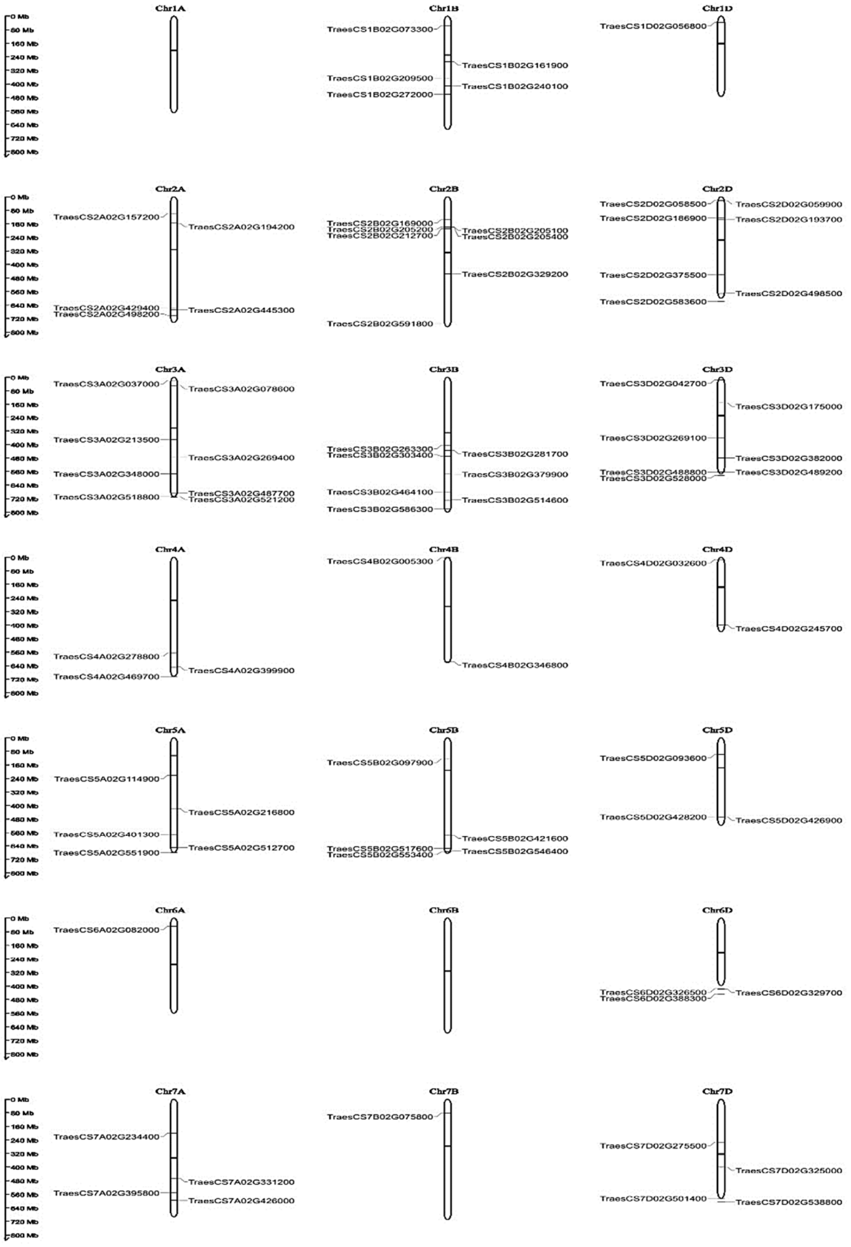

Figure S6. Chromosome location of genes co-expressed with *TaRSL4* under tissue-specific RNA-seq network.

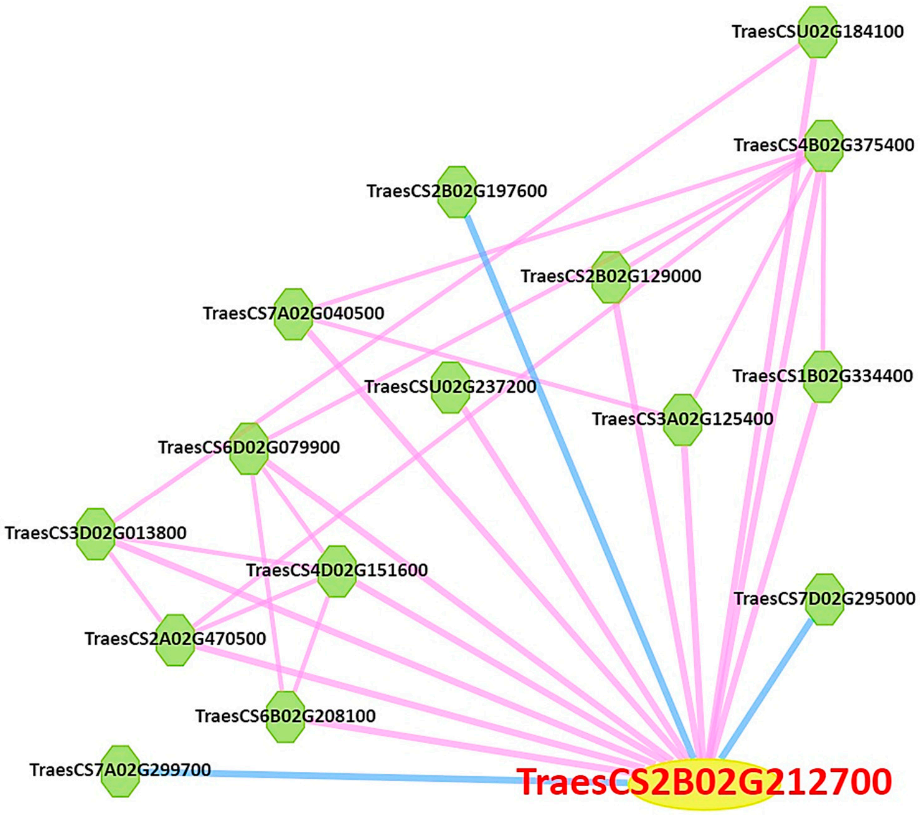

Figure S7. Stress-specific RNA-seq network of *TaRSL4* and its co-expressed genes. Pink interaction lines indicate positive co-expression relationship with target protein whereas blue lines indicate negative co-expression relationship with target protein.

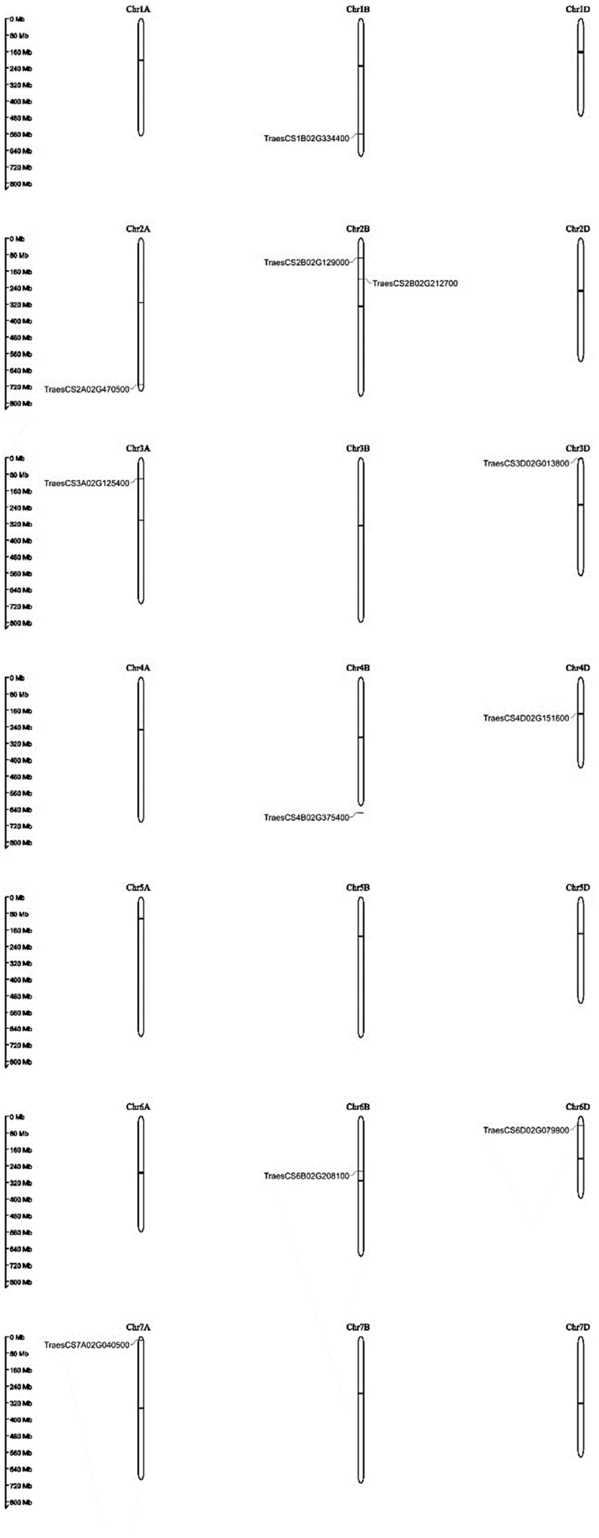

Figure S8. Chromosome location of genes co-expressed with *TaRSL4* under stress-specific RNA-seq network.
